# Brand Experience and Customer Loyalty in Dentistry: The Role of Perceived Brand Authenticity

**DOI:** 10.1155/2023/2541243

**Published:** 2023-11-24

**Authors:** Ghaith Al-Abdallah, Jegr Ababakr

**Affiliations:** ^1^University of Kurdistan Hewlêr, Erbil, Iraq; ^2^University of Liverpool, Liverpool, UK

## Abstract

This study investigates the impact of brand experience (BE) on customer loyalty (CL) and examines the possible mediating effect of brand authenticity (BA) and the moderating effect of frequency of visits on the original relationship between BE and CL in dental practices. A descriptive, deductive, and quantitative research methodology was applied, with a self-administrated survey questionnaire over a cross-sectional convenience sample from dental clinics and centers in Erbil, Sulaymaniyah, and Duhok (Iraqi Kurdistan). Data were collected in July and August 2022. SPSS AMOS 26 was used for analyzing 952 patients' responses. The results indicate that BE has a statistically significant positive effect on CL. However, only affective experiences, cognitive experiences, and behavioral experiences have a statistically positive effect on CL. BA has a significant direct mediation effect on the original relationship between BE and CL. In addition, a greater frequency of dental visits improves the BE and impacts CL. Discussion, recommendations, and future research orientation are provided.

## 1. Introduction

The dental care industry is rapidly evolving and increasingly defying traditional classifications. Given the unpredictability of healthcare industry development as a whole, which is experiencing the opening of new markets and the increased entry of large corporations, dental practices are attempting to develop diverse marketing strategies, including service quality improvement and relationship marketing [1]. In modern dentistry, it is essential to adapt marketing strategy to the current market environment and market analysis, all of which ultimately depend on consumer perceptions and preferences [2]. The health production function model, which dates back to Becker [3], is one of dentistry's most renowned demand models, but it is no longer efficient in explaining market changes. This model assumes that health is the ultimate goal of human desire, whereby individuals choose services due to the necessity of maintaining their oral health. While this might have been the case in the past, where the dentist's role was to treat toothaches and alleviate pain, it is estimated that today, more than 90% of services provided by dentists are discretionary (e.g., cosmetic treatments), which means that very few dental services are directly associated with the mere elimination of pain and suffering associated with the teeth [4].

The majority of dental services may be increasingly viewed as elective by consumers, and the increase and shift in demand for dental practices is leading to new trends in the dental market [5], thereby increasing pressure on dental practices to brand themselves in authentic and appealing ways [6]. Dental practices (centers and clinics) must gain a competitive edge with the aim of increasing their market share to maintain their position in a competitive business environment [7, 8]. In the USA, the marketing expenditure of dental centers increased from USD 29 million in 1997 to USD 114 in 2008 and then to over USD 254 by 2016 [9]. The application of dental marketing has shifted from promotion to branding [10]. Once dentists accept that their dental practice represents a business, they begin to make a series of investments both in the human competence represented by the health and auxiliary personnel, as well as in the latest equipment and materials to reflect their own identity [11, 12].

Transforming dental practices into brands results in changes that add value to the practice for both patients and practitioners. Brand management is generally considered to be one of the prime ways of gaining a competitive edge in any industry [13]. The experience provided by the dental practices is regarded as an essential element for the success of branding, including maintaining existing consumers and attracting new ones [7]. Brand experience (BE) is considered to be the most important intangible asset, especially for brands that interact with a substantial number of consumers annually [14–16]. Competition has become very strong in many dental markets, and BE helps determine customer satisfaction and to position the brand in the market [17–19]. Therefore, it is necessary to establish a powerful brand that focuses on providing a more satisfying customer experience that not only meets but exceeds consumer expectations, which may lead to customer loyalty (CL) [16].

CL can be straightforwardly defined as the behavioral intention of customers to repurchase/reuse products from a particular brand [20]. Loyalty is the most pertinent concept to dental practices nowadays due to the concern to retain customers. Studies have shown that increasing CL by 5% could result in increasing profit by up to 95% [21, 22]. Moreover, in response to service homogenization, a growing quest for authenticity in services can be observed [23, 24]. The concept of brand authenticity (BA) has gained many marketers and researchers' attention in the modern era [25–27]. BA reflects “a subjective evaluation of genuineness ascribed to a brand by consumers” [28]. Previous studies investigated the relationship between branding and CL have found that BE has a direct or indirect impact on CL in a variety of contexts [29–35]. However, there is a lack of similar studies between BE and CL in the dentistry industry. Moreover, literature that examined the relationship between BE and BA is limited [36]. Considering the increased importance of BA and its benefits for the practitioners, the present study develops a model to examine the possible mediating role of perceived brand authenticity (PBA) in dental practices to help bridge the gap found in literature about BA in general and in the dentistry industry.

## 2. Literature Review

### 2.1. CL

Oliver [37] defines loyalty as a customer's dedication or commitment to repurchase the brand of product and service over time, despite the presence of marketing efforts and changing situations that could influence customers to change their minds. In general, CL has behavioral and attitudinal dimensions [38]. Behavioral refers to purchasing and repurchasing intention to a particular brand, while attitudinal refers to how customers are associated with a particular brand [39]. Kang [40] states that behavioral loyalty pertains to the way in which customers repurchase the same product without thinking or making judgment about it. On the other hand, attitudinal loyalty is thought to be a psychological dedication towards a brand that the customer believes the brand is unique [41]. CL is a construct with multiple dimensions, and previous studies on loyalty have generally focused on components such as perceived value, brand trust, and customer satisfaction [41–49]. However, an emerging body of research on brand loyalty has increasingly adopted an integrated approach spanning “Willingness to pay more,” “word of mouth” (WOM), and “repurchase intentions” (RPI) [30, 34, 50, 51], and this more comprehensive paradigm is adopted in the current study.

### 2.2. BE

BE is a concept that was introduced by Schmitt [52] into the experiential marketing literature and whose importance has increased in recent times [53]. Experience occurs directly when shopping, buying, and consuming and indirectly when interacting with media, including traditional and electronic media [54]. BE is regarded as an outcome of customer perceptions resulting from any interaction between the customer and the brand, such as dealing with the staff [55], and feelings, opinions, and beliefs that customers build during their various forms and experiences of engagement with a brand [56]. Brakus et al. [29] viewed BE as an internal response by customers to sensation, including affective, cognitive, and behavioral responses. In this context, BE can be assessed as subjective internal and behavioral responses of consumers associated with stimuli that are a part of a brand. BE thus consists of four dimensions (subconceptual constructs), namely sensory experience, affective experience, cognitive experience, and behavioral experience [29], which are adopted in this study, as adumbrated below:

1. Sensory experience refers to the five human senses that are stimulated as the result of customer and brand interactions.
2. Affective experience refers to customer's feelings toward a brand and the emotional relationship that connects them [57].
3. Cognitive experience refers to the power of a brand to intellectually engage with customers and make them think analytically and imaginatively [58].
4. Behavioral experience refers to a physical activity that consumers engage in when interacting with a brand and the impact of this interaction on customer lifestyle and future behavior [59].

Related interactions between dental practices and consumers can be on several levels, and their outcomes can create a set of ties and bonds through sensory, affective, cognitive (i.e., intellectual), and behavioral experiences and perceptions [35]. Generally, brands that provide excellent experiences are differentiated from other brands and are more favored by consumers, which is reflected in increased brand loyalty, repurchase intention, actual repeat purchases, and recommending the brand to others (i.e., WOM) [60]. Previous studies have shown that good BE has strengthened brand loyalty through several dimensions, such as trust, satisfaction, love, emotional attachment, sense of quality, brand positioning, customer engagement, and customer happiness [34, 35, 50, 61–71].

Based on the reviewed literature on BE, the first main hypothesis and subhypotheses are posited below:

  Ha1: There is a direct significant effect of BE on CL.
  Ha1.1: There is a direct significant effect of sensory experience on CL.
  Ha1.2: There is a direct significant effect of affective experience on CL.
  Ha1.3: There is a direct significant effect of cognitive experience on CL.
  Ha1.4: There is a direct significant effect of behavioral experience on CL.

### 2.3. BA

Authenticity is an important element of any successful brand [72], and customers are now more concerned about the true identity of brands through clear, real, and accurate information than ever before [33]. It is insufficient for companies to develop authentic brands and products *per se*; the object must also be perceived as authentic by the brand's target consumers [73]. Akbar and Wymer [74] defined BA as “the degree to which a brand is considered original and genuine, meaning it is unique and not derivative, and truthful to what it claims to be.” In the same line, PBA has been defined as “the extent to which consumers perceive a brand to be faithful and true toward itself and its consumers” [75]. In most cases, purchasing decisions are made based on the perceived authenticity of brand offerings in terms of the functionality and quality of products (among other factors) [76].

Bruhn et al. [77] conceptualized PBA into four dimensions: continuity (stability, endurance, and consistency), reliability (credibility, trustworthiness, and delivering and keeping what it promises), originality (uniqueness and innovative), and naturalness (genuineness, realness, harmony, and lack of artificiality in design). This model is among the most widely used in literature and, therefore, was adopted for this study to measure PBA.

Efforts have been made to study the consequences of PBA in different areas and contexts, such as brand auras [78], brand trust [79, 80], product perceptions [81], brand attachment [75], brand choice intentions [82], brand attitude [83, 84], word-of-mouth [33, 75], and purchase intentions [28]. While the previous studies highlighted the mediating role that PBA might have, none of them considered the health sector in general or dental practice in particular. Given that dental practices are officially becoming brands, it is very important to understand the mediating role of PBA on the original relationship between BE and CL. Accordingly, the second hypothesis is formulated:

  Ha2: PBA mediates the original relationship between BE and CL.

### 2.4. The Role of Frequency of Visits (FoV)

As explained previously, BE results from customer interactions with the brand; accordingly, the frequency and duration of such interactions are assumed to directly moderate customer perceptions and affect future intentions. Mohsen et al. [85] examined the possible moderating effect of FoV in the healthcare context and demonstrated that it does indeed moderate the relationship between service quality and customer engagement. The results indicated a significant difference between respondents regarding the relationship between control on customer engagement because of their FoV. As FoV means more or less engagement with the BE, it would be interesting to examine the possible moderation role FoV plays in the relationship between BE and CL in the dental context. Accordingly, the third hypothesis is posited for investigation:

  Ha3: FoV moderates the original relationship between BE and CL.

### 2.5. Research Framework

**Figure 1:**
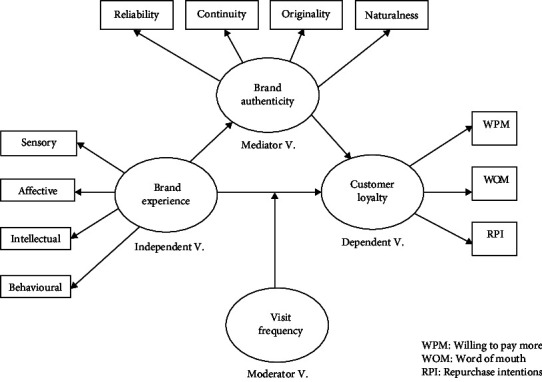
Research conceptual model (source: authors).

The aim of this study is to establish the relation between BE (independent variable) and CL (dependent variable) mediated by BA (mediator variable) and moderated by FoV (moderating variable) within the dental practice context. Following the above literature review and the formulated research hypotheses, the conceptual model shown in Figure 1 was formed.

## 3. Research Methodology

As the study is trying to identify the relationship between BE and CL as well as the mediating role of BA and the moderating influence of FoV, the study follows a descriptive methodology, which is the most applicable approach for the purposes of this study context. Within this type of research, researchers do not have any control over the variables and can only report what is going on (without controlling the variables) [86]. The study follows a deductive approach, whereby theory and hypothesis development based on the literature is used to test relationships between studied variables [87]. The approach involves quantitative data, and a large sample is involved to ensure the generalization of the results. This study uses a survey strategy, which is commonly associated with deductive and quantitative research, whereby limited and structured questions are used, and results are structured in a quantified manner [87]. A questionnaire is the main tool to collect primary data in this study. SPSS AMOS 26 was used to test the hypotheses, with the application of a variance-based structural equation model (SEM) and partial least squares (PLS) to simultaneously model relationships between multiple dependent and independent variables.

### 3.1. Research Instrument

A self-administered questionnaire developed by the researchers based on previous studies and professionally translated into Kurdish and Arabic to accommodate all possible respondents was used as the primary method of data collection was applied as the primary method of data collection. Before the data were collected, the questionnaire was validated through conducting a pilot study with 47 respondents from several dental practices to verify that the statements were clear and understandable, that the language was clear and precise, that the nature of the responses to the statements coincides to the true intent, and that the questionnaire measures what it is meant to be measured. Validity and reliability analyses were carried out as well. Based on the results of the pilot study, necessary changes were made, and the final questionnaire was ready for distribution (Appendix A).

The questionnaire comprises four parts dedicated to measuring the independent variable BE, the dependent variable CL, the mediating variable PBA, and demographic characteristics and the moderating variable FoV. Items in the first three parts are answered with five-point Likert scales (ranging from 1 = *strongly disagree* to 5 = *strongly agree*), while the fourth part used multiple-choice questions.

The measurements of the independent variable BE and its indicators (dimensions) were developed based on previous studies [29, 34, 88]. The scale consists of 16 items, with four items each for the sensory, affective, cognitive, and behavioral experience indicators.

The measurements of the dependent variable CL and its indicators were adapted from previous studies [48, 49, 51]. The scale consists of 11 items, comprising 3 for WPM, 4 for WOM, and 4 for RPI.

The measurements of the mediator variable PBA and its indicators were adapted from Bruhn et al. [77]. The scale consists of 13 items (4 for reliability, 3 for continuity, 3 for originality, and 3 for naturalness).

A cover letter describing the type, nature, and purpose of the research and assuring anonymity and voluntary participation was provided in all three languages, including the right to withdrawal and assurance that any decisions would not affect dental or other healthcare services received and potential participants' statutory rights.

### 3.2. Study Population, Sample, and Recruitment

The studied population comprised patients visiting dental practices for dental treatments in the most populous cities of Iraqi Kurdistan; this population cannot be definitively estimated due to the lack of any official records (and the politicized and contentious nature of population studies in the region). However, since most people need some kind of dental services, the research population was projected as the majority of the total population of Iraqi Kurdistan, which was estimated to be 6.2 million as of 2020 [89]. For such a large population, Cochran's [90] formula was used to estimate the cross-sectional sample size to increase the accuracy of the representation, with the application of a 3% margin of error [91]. Accordingly, the sample representation of this research is about 1,067.

Due to the fact that full records of dental patients cannot be obtained, the study used a nonprobability, convenience sampling technique. In Iraqi Kurdistan, there are 899 dental clinics (each with up to three chairs) and 346 dental centers (with 4–12 chairs); all are private practices, distributed as follows: Erbil has 261 clinics and 123 centers; Sulaymaniyah has 496 clinics and 214 centers; and Duhok has 142 clinics and 9 centers [92]. The cost of dental services among these clinics and centers can vary depending on the available treatments and the location, the reputation of the dental clinic, and the experience and qualifications of the dental practitioner. Research assistants visited dental practices that were interested in collaborating with the researchers and assisting in collecting data, where three research assistants covered Erbil, four covered Sulaymaniyah, and two covered Duhok. The assistants are master's students in their second year who have already passed the capstone module in research methods at the postgraduate level. In addition, the assistants have received training related directly to the study in hand, including the research questions, objectives, and hypotheses, in order for them to understand the importance of the study and the role they will be playing in the research process before training them on the data collection. After that, the assistance received hands-on training to ensure that the research assistants have a good understanding of the questionnaire administration process, providing them with opportunities to practice data collection procedures and receive feedback on their performance under the supervision of the researchers. Personally administered questionnaires were used to collect the data from available patients in the visited practices who consented to anonymously participate in the research. Data were collected in July and August 2022. The targeted 1,067 responses were obtained. After the screening and filtration process, 952 valid and usable responses were statistically analyzed.

Table 1 presents the demographic variables of the study sample, indicating that most respondents were females (62.8%), and the majority of participants were aged 18–33 years' old (82.6%). In terms of their highest level of education, most had a Bachelor's degree (57.1%), while 21.8% had a postgraduate degree. The largest cohort (over a quarter) (26.9%) had a monthly income of USD 500–1,000, while almost a fifth had income of USD 1,501–2,500 (19.3%) or USD 1,001–1,500 (18.7%). The majority of respondents reported an annual FoV of one to two visits.

## 4. Results

### 4.1. Confirmatory Factor Analysis (CFA)

To evaluate the correlations between the study variables, namely the factor loadings, CFA was utilized. The results are presented in Table 2, along with the composite reliability (CR) and convergent validity values. For the evaluation of discriminant validity, the heterotrait–monotrait ratio of correlations (HTMT) criterion was employed, and the results are outlined in Table 3.

In Table 2, it is evident that the loadings of all items fall within the range of 0.655–0.887. This aligns with the recommended factor loading threshold of 0.50 or higher, and ideally, 0.70 or higher, as stated by Bollen [93], indicating the acceptability of the results.

To establish convergent validity in factor loadings, CR and average variance extracted (AVE) were examined. The results reveal that the CR values, spanning from 0.815 to 0.901, surpass the 0.7 threshold, which signifies robust internal consistency. Additionally, the AVE values, failing between 0.526 and 0.696, exceed the 0.50 benchmark, thus justifying the utilization of the construct. Consequently, all latent variables fulfill the requisite criteria for establishing convergent validity [94].

Table 3 illustrates that all the HTMT values obtained are below 0.85, which suggests the absence of any issues related to discriminant validity, as HTMT values below 0.90 establish discriminant validity between reflective constructs [95]. Based on the results, no collinearity problems among the latent constructs (multicollinearity) and no overlapping items from the respondents' perceptions in the affected constructs were detected.

**Figure 2:**
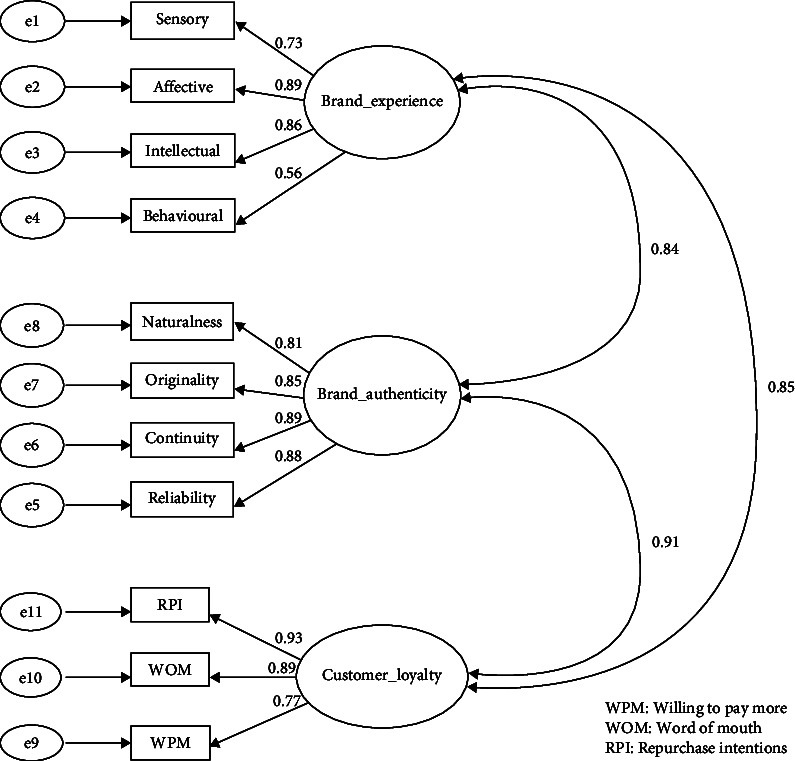
Final best-fitting CFA model (source: SPSS AMOS 26).

Based on the results of Tables 2 and 3, the final best-fitting model is presented in Figure 2.

### 4.2. Goodness of Fit

To evaluate the goodness of fit for the model, a range of indicators are referred to, including standardized root mean squared residual (SRMR), comparative fit index (CFI), Tucker and Lewis's index of fit (TLI), normed fit index (NFI), and root mean square error of approximation (RMSEA). The results are presented in Table 4, which shows that the value of SRMR is less than 0.08, indicating an excellent model fit [96]. The CFI value is greater than 0.95, indicating a good fit for the model [97]. The TLI value is greater than 0.95, indicating a good fit [98]. The NFI value is greater than 0.95, also indicating a good fit for the model [96]. The RMSEA is less than 0.1, which indicates a reasonable fit for the model [99]. As indexes suggest a sufficient fit of the model to the current data, the hypothesized model is fitted.

### 4.3. Testing the Hypotheses

**Figure 3:**
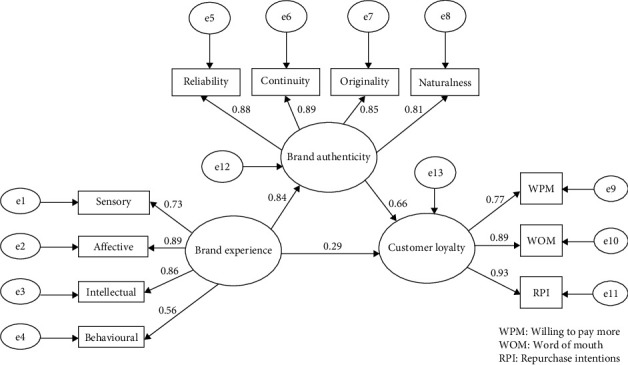
The SEM model for the hypotheses (source: SPSS AMOS 26).

To test the hypotheses of this research, variance-based SEM and PLS were used to model relationships between multiple dependent and independent variables at the same time, which is needed in this research. The results are presented in Figure 3 and are explained in the subsections below.

#### 4.3.1. Testing the First Hypothesis

  Ha1: There is a direct significant effect of BE on CL.

The first hypothesis was tested by SEM, using the significance threshold defined by Byrne [100]. The results are presented in Table 5.

 
*Ha1.1: There is a direct significant effect of sensory experience on CL*.

According to the regression weights, sensory experience has an insignificant effect on CL; the path is insignificant since the critical ratio value is less than 2, and the *p*-value (0.170) is greater than 0.05. Accordingly, the first alternative subhypothesis is rejected, indicating that sensory experience has an insignificant effect on CL at *α* ≤ 0.05.

 
*Ha1.2: There is a direct significant effect of affective experience on CL*.

Affective experience has a significant effect on CL; the path is significant since the critical ratio value is greater than 2, and the *p*-value ( ^*∗*^ ^*∗*^ ^*∗*^) is less than 0.05. Accordingly, the second alternative subhypothesis is accepted, indicating that affective experience has a significant positive effect on CL at *α* ≤ 0.05, with an effect size of 0.187.

 
*Ha1.3: There is a direct significant effect of cognitive experience on CL*.

Cognitive experience has a significant effect on CL; the path is significant since the critical ratio value is greater than 2, and the *p*-value ( ^*∗*^ ^*∗*^ ^*∗*^) is less than 0.05. Accordingly, the third alternative subhypothesis is accepted, indicating that cognitive experience has a significant positive effect on CL at *α* ≤ 0.05, with an effect size of cognitive 0.131.

 
*Ha1.4: There is a direct significant effect of behavioral experience on CL*.

Behavioral experience has a significant effect on CL; the path is significant since the critical ratio value is greater than 2, and the *p*-value ( ^*∗*^ ^*∗*^ ^*∗*^) is less than 0.005. Accordingly, the fourth alternative subhypothesis is accepted, indicating that behavioral experience has a significant effect on CL at *α* ≤ 0.05, with an effect size of 0.151.

Affective experience has the highest effect size (0.187) on CL, followed by behavioral experience and then cognitive experience, with effect sizes of 0.151 and 0.131, respectively.

The totality of BE has a significant positive effect on CL, and the path is significant, since the critical ratio value is greater than 2, and the *p*-value ( ^*∗*^ ^*∗*^ ^*∗*^) is less than 0.05. Accordingly, the first main alternative hypothesis is accepted, indicating that BE has a significant positive effect on CL at *α* ≤ 0.05. The effect size of BE on CL is 0.293. Moreover, the *R*^2^ value, which indicates the ability of the independent variable to explain changes in the dependent variable, shows that BE can explain 71.6% of the variation in CL, as the *R*^2^ value is 0.716.

#### 4.3.2. Testing the Second Hypothesis

  Ha2: PBA mediates the original relationship between BE and CL.

The second hypothesis is tested by SEM. The results are presented in Table 6, showing that BE has a significant effect on PBA, since the critical ratio value is greater than 2 here and the *p*-value (0.000) is less than 0.05. The effect size of BE on PBA is 0.838. PBA has a significant direct effect on CL, since the critical ratio value is greater than 2 here and the *p*-value (0.000) is less than 0.05. The effect size of PBA on CL is 0.664. Accordingly, the second alternative hypothesis is accepted, indicating that PBA has a significant direct mediation effect on the original relationship between BE and CL at *α* ≤ 0.05. The direct effect of BE on Cl increased from 0.293 to 0.556 due to the mediating presence of PBA. In the same line, the *R*^2^ value, which indicates the ability of the independent variable (BE) to explain changes in the dependent variable (CL), increased from 71.6% to 85.2% due to the mediating effect of PBA.

#### 4.3.3. Testing the Third Hypothesis

  Ha3: FoV moderates the original relationship between BE and CL.

The third main hypothesis is tested through multiple-group SEM analysis using AMOS, and the results are presented in Table 7, showing that the *χ*^2^ value (52.591) is significant, since the *p*-value (0.000) is less than (0.05). This means that there are significant differences between the different numbers of visits by year groups; therefore, the FoV per year has a significant moderating effect on the original relationship between BE and CL.

The results indicate that prolific FoV (i.e., for the third group, who attend seven times and more) had the biggest effect size on the original relationship between BE and CL, with an effect size equal to (1.618), followed by the second group (three to six times), and then the first group (one to two times), with effect sizes of 0.667 and 0.228 (respectively).

## 5. Conclusion and Recommendations

### 5.1. Main Outcomes

According to the findings of this study, BE has a considerable and direct significant positive impact on CL, which is consistent with Brakus et al.'s [29] research on the relationship between BE and CL but contrary to the finding of Francisco-Maffezzolli et al. [31] that BE only *indirectly* affects CL. Among the indicators of BE, CL is not significantly impacted by sensory experience, while affective experience, cognitive experience, and behavioral experience were found to have significant impacts on CL, and affective experience was the most influential. This finding reflects that dental care is a very delicate, sensitive, and emotional service industry, and strong emotional attachment from clients toward practices might come from the sympathetic and professional approaches by dentistry professionals and the feeling of being in safe hands. These results are in line with the findings of Ferreira et al. [35] on this point, as they also reported that affective experience has the strongest effect among the dimensions of BE.

Results of this study also indicated that behavioral experience and cognitive experience have impacts on CL. Behavioral experience, which is derived from physical actions during treatment and how dental practices influence customer behavior through changing some habits, seems to be important in achieving CL. The cognitive experience derived from engaging customers with the treatment plan and making them think about how the equipment and the treatment works has an important role in influencing CL as well. The cognitive experience triggers customers' curiosity in particular phenomena of dentistry services, such as high-quality and modern dental equipment and practices.

Finally, unlike affective, behavioral, and cognitive experience, this study found that sensory experience was lacking any significant impact on CL. A possible explanation for this finding could be in the context of dental industry, wherein sensory experience does not form a more substantive part of customer perception as the human five senses may be preoccupied with medical dentistry paraphernalia in surgeries (e.g., unpleasant detergent odors, and medical materials and substances), unlike the pleasant sensory experiences encountered in conventional retail contexts (e.g., malls and restaurants). For instance, Ong et al. [34] concluded that all dimensions of BE (including sensory) have significant impacts on CL in the context of the restaurant industry, and Ferreira et al. [35] found the sensory experience to be significant on CL in the retail fashion industry. This might suggest that the effect of BE dimensions varies in different industries and contexts, consistent with examinations of the impacts of BE on brand loyalty in different contexts, directly and indirectly, through different mediating variables [32, 62–64, 68]. These studies either found that BE has a significant positive direct effect on CL or indirectly through the mediating variables.

According to the current study, CL is significantly mediated by PBA. It seems that for customers of dental practices, it is important to know the foundation of dental clinics or centers, in terms of their values, the reliability and credibility of their treatment, their naturalness, and their originality and uniqueness. Dental practice is a very sensitive service, and engendering and maintaining CL requires that services are original and authentic, communicating with clients honestly and transparently, delivering credible services, and making logical and reliable services. This result is consistent with Safeer et al. [26] on the mediating role of PBA in the relationship of BE and CL. In addition, Yildiz and Ulker-Demirel [33] found that there is a positive direct effect of PBA on WOM (one of the loyalty dimensions). Eggers et al. [79] also found that PBA has a significant impact on brand trust. PBA is a multidimensional construct; hence, different authors used different dimensions to study it [101]; however, regardless of the particular dimensions used, PBA undoubtedly plays a significant mediating role between branding and CL.

Another main result of this study is that the FoV per year has a moderating effect on the original relationship between BE and CL. The study concludes that the more frequently customers visit dental practices, the greater the moderating effect size of FoV. The more the customers are engaged with the practice, the more their BE affects their loyalty. This is in line with Mohsen et al. [85], who found that more visits to the healthcare provider led to more customer engagement and, therefore, loyalty. It seems that increased FoV to a particular dental practice engenders an emotional and affective connection with the practice, which results in developing behavioral loyalty into attitudinal loyalty.

### 5.2. Managerial Implications

Based on the results of the study, it is important that dental care providers pay more attention to dimensions of band experience, such as affective, cognitive, and behavioral experience, in order to achieve CL. Special attention should be paid to the affective experience. Dental practices should consider patients' concerns effectively and professionally to create a strong emotional attachment and make them feel safe, confident, and attached to the practices and the services it provides. Dental practices should engage clients in a way that encourages them to change negative behaviors (e.g., smoking, consuming sugary beverages and sweets, etc.) or promote the adoption of positive ones (e.g., regular flossing and brushing of teeth, regular checkups, eating healthy food, etc.). Therefore, dental practices should not only provide dental services but also provide dental education dialogs, which can foster CL.

Cognitive experience is also found to be one of the factors that affect CL. Therefore, the study recommends that dental practices should intellectually and clearly explain diagnosis and treatment plans to patients and provide information about dental procedures. In addition, the treatment booking and payment processes should be clear, smooth, and well-organized. Such simple service improvements can create an excellent cognitive experience that intrigues patients and encourages CL.

In addition, since PBA has a significant positive effect on the original relationship between BE and CL, it is essential that the dental services to provide reliable, credible, and consistent services, with uniqueness manifest in original and natural identity, to create a competitive advantage. Finally, the study recommends that dental practices should facilitate and encourage more visits of their clients to increase the level of exposure to the experience received, which will positively affect CL formation.

### 5.3. Study Limitations and Future Research Directions

The sample of this study was obtained from the main cities in Kurdistan; consequently, it has geographical limitations and does not represent the whole population of Iraq. Furthermore, the population was notably well-educated and relatively affluent, with 78.9% having a degree or postgraduate qualifications and 60.2% earning over USD 1,001 per month; the 12.8% who earned less than USD 500 per month presumably included some unemployed customers, but the findings of this study cannot be considered representative of the average resident of Iraqi Kurdistan due to the sociodemographic profile of participants. As participants were not purposively recruited in terms of their income/employment status and educational level, it is likely that these findings reflect barriers to accessing dental care among residents; although the provision of and access to dental services has increased greatly since 2003, barriers still remain, particularly for certain disadvantaged groups, with a lot of dental visits being undertaken in private afternoon clinics [102]. Future research should examine the same research model in other Iraqi governance and regions and across a broader spectrum of dental clients in terms of sociodemographic characteristics in order to provide more generalizable results.

The same research can also be conducted in other markets for the same purpose. Moreover, the same research can be conducted on other healthcare sectors and service providers (e.g., pharmacies, hospitals, medical centers and clinics, medical laboratories, etc.) to compare the results and draw general conclusions.

Further qualitative studies are required to further investigate the findings of this study to understand the reasons that lead to such results and also exploring customer buying behavior. It is important to test the relationship of other branding concepts, such as brand attachment, brand love, brand trust, and brand satisfaction, with customer behavioral and attitudinal loyalty. Moreover, BE and BA are multidimensional concepts, and this study selected a specific set of dimensions to measure them; other dimensions could result in different results and would deepen the understanding of the relationship between the variables. Finally, the study used BA as a mediator variable; future studies are encouraged to incorporate other brand concept mediators for the current theoretical model. This is important to establish the relationship between BE and CL with multiple mediating variables.

## Figures and Tables

**Table 1:** Participant characteristics.

Variable	Category	Number	Percent
Gender	Male	345	37.2
	Female	598	62.8
	Total	951	100

Age (years)	<18	10	1.1
	18–23	392	41.2
	24–33	394	41.4
	34–49	138	14.5
	50+	18	1.9
	Total	952	100

Education level	High school or less	96	10.1
	Diploma degree (institution)	104	10.9
	Bachelor's degree	544	57.1
	Postgraduate degree	208	21.8
	Total	952	100

Monthly income (USD)	<500	122	12.8
	500–1,000	256	26.9
	1,001–1500	178	18.7
	1,501–2,500	184	19.3
	2,501–3,999	126	13.2
	4,000+	86	9
	Total	952	100

Number of yearly dental visits	1-2	572	60.1
	3–6	238	25
	7+	142	14.9
	Total	952	100

Source: SPSS AMOS 26.

**Table 2:** Confirmatory factor analysis results (factor loading).

Latent variable	Indicator	FL	FLS	AVE (>0.50)	CR (>0.70)
Brand experience	Sensory experience	0.655	0.429	0.526	0.815
	Affective experience	0.811	0.658		
	Cognitive experience	0.767	0.588		
	Behavioral experience	0.655	0.429		

Brand authenticity	Reliability	0.853	0.728	0.696	0.872
	Continuity	0.849	0.721		
	Originality	0.849	0.721		
	Naturalness	0.784	0.615		

Customer loyalty	Willingness to pay more	0.742	0.551	0.696	0.901
	Word of mouth	0.867	0.752		
	Repurchase intentions	0.887	0.787		

*Note*: FL = factor loading, FLS = factor loading squared, AVE = average variance extracted, CR = composite reliability. Source: SPSS AMOS 26.

**Table 3:** HTMT analysis.

	Brand experience	Brand authenticity	Customer loyalty
Brand experience			
Brand authenticity	0.849		
Customer loyalty	0.828	0.815	

Source: SPSS AMOS 26.

**Table 4:** Goodness-of-fit statistics for three-factor CFA model.

Model tested	*χ* ^2^	SRMR	CFI	TLI	NFI	RMSEA
Model performance	193.93	0.036	0.965	0.953	0.956	0.089
Criterion for goodness of fit	—	—	≥0.90	≥0.90	≥0.90	≤0.10

Source: SPSS AMOS 26.

**Table 5:** Structural equation modeling regression weights.

Model			Estimate	S.E.	C.R.	*p*	Effect	*R* ^2^
Customer loyalty	←	Sensory experience	−0.034	0.025	−1.373	0.170	−0.045	—
Customer loyalty	←	Affective experience	0.155	0.038	4.116	^*∗*^ ^*∗*^ ^*∗*^	0.187	—
Customer loyalty	←	Cognitive experience	0.115	0.029	3.905	^*∗*^ ^*∗*^ ^*∗*^	0.131	—
Customer loyalty	←	Behavioral experience	0.132	0.028	4.635	^*∗*^ ^*∗*^ ^*∗*^	0.151	—
Customer loyalty	←	Brand experience	0.571	0.120	4.754	^*∗*^ ^*∗*^ ^*∗*^	0.293	0.716

Source: SPSS AMOS 26.

**Table 6:** Structural equation modeling regression weights for the mediating effect of perceived brand authenticity.

Model			Estimate	S.E.	C.R.	*p*	Effect	*R* ^2^
Perceived brand authenticity	←	Brand experience	1.368	0.114	12.021	^*∗*^ ^*∗*^ ^*∗*^	0.838	0.701
Customer loyalty	←	Brand experience	0.571	0.120	4.754	^*∗*^ ^*∗*^ ^*∗*^	0.293	0.716
Customer loyalty	←	Perceived brand authenticity	0.794	0.076	10.451	^*∗*^ ^*∗*^ ^*∗*^	0.664	—
Customer loyalty ← perceived brand authenticity ← brand experience			—	—	—	—	0.556	0.852

*Note*: S.E. = standard errors of the regression weights, C.R. = critical ratio, *p* = *p*-value. Source: SPSS AMOS 26.

**Table 7:** Multiple-group SEM analysis results frequency of visits.

Model	DF	CMIN	*p*	NFI delta-1	IFI delta-2	RFI rho-1	TLI rho-2
Structural weights	22	52.591	0.000	0.009	0.009	0.000	0.000
No. of visits				Regression weights			
1-2	BE	→	CL	0.228			
3–6	BE	→	CL	0.667			
7+	BE	→	CL	1.618			

*Note*: Nested model comparisons (assuming model unconstrained to be correct). Source: SPSS AMOS 26.

## Data Availability

Data are stored on a secured external hard disc with the first author, and it will be available upon request.

## Appendix

### A. Final Questionnaire

#### A.1. Brand Experience

**Table:**

No	Statements	Strongly agree	Agree	Neutral	Disagree	Strongly disagree	I do not know
	Sensory experience						

1	The interior design and decorations of this dental practice appeal to my visual sense	1	2	3	4	5	6

2	The background music in the waiting room and the dental room appeals to my sense of hearing	1	2	3	4	5	6

3	The odors of this dental practice appeal to my smell sense	1	2	3	4	5	6

4	I find this dental practice interesting in a sensory way						

	Affective experience						

5	The sympathetic attitude of the staff creates a strong emotional attachment to this practice	1	2	3	4	5	6

6	The level of professionalism in this dental practice makes me feel confident	1	2	3	4	5	6

7	Considering my concerns to the dentist makes me feel secured	1	2	3	4	5	6

8	I have positive feelings for this dental practice	1	2	3	4	5	6

	Cognitive experience						

9	The administration processes in this dental practice intrigue me	1	2	3	4	5	6

10	The modern equipment used by this dental practice stimulates my curiosity	1	2	3	4	5	6

11	Explaining my case diagnosis by the dentist engage me intellectually	1	2	3	4	5	6

12	Going over my treatment plan relates to my problem-solving skills.						

	Behavioral experience						

13	I engage in physical actions as part of my treatment when I visit this dental practice	1	2	3	4	5	6

14	I must move between different sections of the practices to get my treatment	1	2	3	4	5	6

15	Visiting this dental practice drives me to change my habits (brushing my teeth, flossing, stopping smoking, etc.)	1	2	3	4	5	6

16	This dental practice results in bodily experiences	1	2	3	4	5	6

#### A.2. Customer Loyalty

**Table:**

No	Statements	Strongly agree	Agree	Neutral	Disagree	Strongly disagree	I do not know
	Willingness to pay more (WPM)						

17	I am willing to pay a higher price for the service of this dental practice than other dental practices	1	2	3	4	5	6

18	I will continue to use the service of this dental practice even if their price increase	1	2	3	4	5	6

19	I will continue to use the service of this dental practice even if other practices run promotional discounts	1	2	3	4	5	6

	Word of mouth (WOM)						

20	I recommended this dental practice to my family and friends	1	2	3	4	5	6

21	I speak positively about this dental practice with others	1	2	3	4	5	6

22	If someone mentions dental practice service, I will immediately suggest this dental practice	1	2	3	4	5	6

23	If someone makes a negative comment about this dental practice brand, I will defend it	1	2	3	4	5	6

	Repurchase intentions (RPI)						

24	I will keep using the service of this dental practice in the future	1	2	3	4	5	6

25	I would still use the service of this dental practice even if I received information about other dental practices' options	1	2	3	4	5	6

26	When I am thinking about dental practice services, I always decide to use the services provided by this dental practice	1	2	3	4	5	6

27	I am committed to this dental practice	1	2	3	4	5	6

#### A.3. Brand Authenticity

**Table:**

No	Statements	Strongly agree	Agree	Neutral	Disagree	Strongly disagree	I do not know
	Reliability						

28	This dental practice makes reliable promises	1	2	3	4	5	6

29	The dental practice delivers what it promises	1	2	3	4	5	6

30	The dental practice's services are credible	1	2	3	4	5	6

31	This dental practice is transparent and honest with its clients	1	2	3	4	5	6

	Continuity						

32	I think this dental practice stays true to itself	1	2	3	4	5	6

33	This dental practice has a clear concept that it pursues	1	2	3	4	5	6

34	The service quality of this dental practice is stable and timeless (i.e., service quality does not decrease over time)	1	2	3	4	5	6

	Originality						

35	This dental practice stands out from other practices	1	2	3	4	5	6

36	I think this dental practice brand is unique	1	2	3	4	5	6

37	This dental practice introduced new and innovative techniques in dental care	1	2	3	4	5	6

	Naturalness						

38	This dental practice does not seem artificial	1	2	3	4	5	6

39	This dental practice gives the impression of being natural	1	2	3	4	5	6

40	I do not feel that this practice is manipulating me	1	2	3	4	5	6

#### A.4. Demographics

**Table:**

In the following, please select the category that best describes you:		
41	Gender	Male
		Female

42	Age (years)	<18
		18–23
		24–33
		34–49
		50+

43	Education level	High school or less
		Diploma degree (institution)
		Bachelor's degree
		Postgraduate degree

44	Monthly income (USD)	<500
		500–1,000
		1,001–1,500
		1,501–2,500
		2,501–3,999
		4,000+

45	Number of yearly dental visits	1–2
		3–6
		7+
